# Repair of Cranial Defects in Rabbits with 3D-Printed Hydroxyapatite/Polylactic Acid Composites

**DOI:** 10.1155/2022/7562291

**Published:** 2022-12-31

**Authors:** Guofeng Fan, Liu Yang, Dong Liu, Yongxin Wang, Wenyu Ji, Tukebai, Hu Qin, Zengliang Wang

**Affiliations:** ^1^Center of Neurosurgery, The First Affiliated Hospital of Xinjiang Medical University, China Ürümqi 830054; ^2^Institute of Radiology, Charité-Universitätsmedizin Berlin, Germany Berlin 10117; ^3^Department of Health Management Center, The First Affiliated Hospital of Xinjiang Medical University, China Ürümqi 830054

## Abstract

**Objective:**

The safety and efficacy of three-dimensional- (3D-) printed hydroxyapatite/polylactic acid (HA-PLA) composites in repairing cranial defects were evaluated in a rabbit experimental model.

**Methods:**

Twelve New Zealand rabbits were selected as experimental subjects. Two holes (A and B), each with a diameter of approximately 1 cm, were made in the cranium of each rabbit. Hole A served as the experimental manipulation, and hole B served as the control manipulation. A 3D-printed HA-PLA composite was used for placement onto hole A, whereas autologous bone powder was used for placement onto hole B. Samples from the experimental holes and the control holes were collected at 30 and 90 days after surgery. The obtained materials were examined in terms of their morphologies and histopathologies and were also subjected to simultaneous hardness tests.

**Results:**

Both the 3D-printed HA-PLA composite and autologous bone powder were able to repair and fill the cranial defects at 30 days and 90 days after surgery. At 30 days after surgery, the microhardness of the area repaired by the HA-PLA composite was lower than that of the area repaired by autogenous bone powder (*p* < 0.01), but neither of these treatments reached the hardness of normal bone at this time (*p* < 0.01). At 90 days after surgery, there was no statistically significant difference in the microhardness of the repaired area from the 3D-printed HA-PLA composite compared with that of the repaired area from autologous bone powder (*p* > 0.05), and there was no statistically significant difference in the hardness of the two repaired areas compared with that of the normal bone (*p* > 0.05). Hematoxylin-eosin staining showed that bone cells in the HA-PLA material in the experimental group grew and were arranged in an orderly manner. Bone trabeculae and marrow cavities were formed on the pore surface and inside of the HA-PLA scaffold, and the arrangement of bone trabeculae was regular.

**Conclusion:**

3D-printed HA-PLA composites can induce bone regeneration, are biocompatible, have the same strength as autologous bone powder, are able to degrade, and are ultimately safe and effective for repairing cranial defects in rabbits. However, further research is needed to determine the feasibility of 3D-printed HA-PLA composites in human cranioplasty.

## 1. Introduction

The use of hydroxyapatite/polylactic acid (HA-PLA) composites as scaffolds in bone generation has recently been investigated and implemented across the world, which has demonstrated that this approach has good biocompatibility and osteoinduction [1, 2]. Zhang et al. [3] applied three-dimensional- (3D-) printed HA-PLA composites into rabbit tibia to construct tissue-engineered bone and obtained efficacious results. Specifically, 3D-printing technology collectively refers to the additive manufacturing and processing technologies that divide 3D images into two-dimensional thin layers and then use a 3D printer to pile up reinforced materials layer by layer to form 3D objects. Compared with properties of traditional manufacturing technologies, 3D-printing technology shortens the production cycle, reduces production costs, and achieves more accurate shaping [4]. With societal progress in recent years, patients' requirements for cranial repairs are no longer limited to brain protection but also include functional- and aesthetic-based repairs. Although cranioplasty has been developed for hundreds of years, there is still no global consensus on the optimal materials for cranioplasty [5]. The feasibility studies of evaluating HA-PLA composite as skull repair material are limited at China and abroad. In the present study, the advantages of 3D-printing technology and HA-PLA composites were combined to explore the safety and efficacy of this approach in repairing rabbit skull defects. Our findings may aid in further determining optimal materials and approaches for skull repair in clinical practice.

## 2. Materials and Methods

### 2.1. Time and Location of the Present Study

The experiment was conducted from March 2017 to January 2019 at the animal experiment center of the First Affiliated Hospital of Xinjiang Medical University and the School of Materials Engineering at Xinjiang University. The experimental protocol was approved by the hospital ethics committee (approval number: IACUC20170315-07).

### 2.2. Materials

#### 2.2.1. Experimental Animals

Twelve healthy adult male and female New Zealand rabbits (2.73 ± 0.21 kg) were selected as experimental animals. The experimental animals were provided by the animal experimental center of the First Affiliated Hospital of Xinjiang Medical University.

#### 2.2.2. Reagents

The following reagents were used: polylactic acid (PLLA; molecular weight =200,000; Shandong Fanggang Co., Ltd); nanohydroxyapatite (nHA; Sigma, USA; diameter<200 nm); paraformaldehyde (Chengdu Kelong Chemical Co., Ltd.); hematoxylin-eosin staining reagent (Nanjing Jiancheng Biological Products Co., Ltd.); and neutral decalcification solution (Zhongshan Jinqiao Biological Company).

#### 2.2.3. Instruments

The following instruments were used: electronic balance (Shanghai Jingtian Balance Factory); small electric drill (Dremel, USA); optical microscope (Olympus BX51, Japan); paraffin slicer (Leica, Germany); and video Vickers hardness tester (mhv-10z, Shanghai Binocular).

### 2.3. Experimental Methods

#### 2.3.1. Preparation of HA-PLA Composites

**Figure 1:**
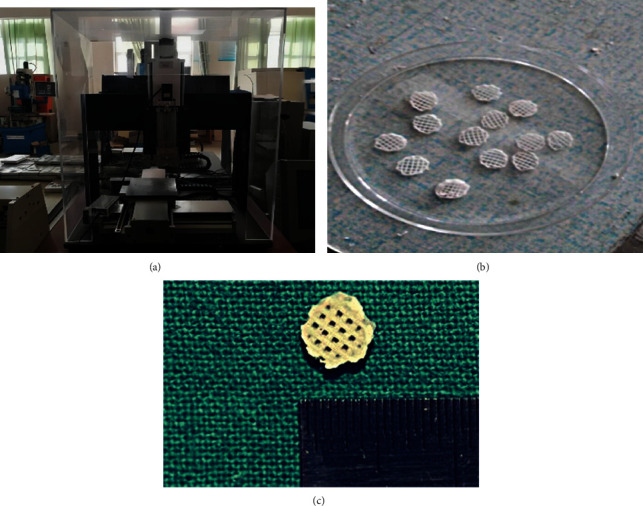
Preparation of HA-PLA composites.

The support material was designed as a cylinder with a diameter of 1 cm and a thickness of 2 mm, and the porosity of the material was set at 60%. The designed 3D model was placed into the 3D printer in STL format (Figure 1(a)). The printing path was adjusted to make the reticular complex. PLA was first fully dissolved in the organic solvent, trichloromethane, in a water bath at 60°C, and then powdered nanometer HA was slowly added in a state of agitation (the mass ratio of HA to PLA was 15 : 85). The ultrasonic wave vibrated for 15 min, and electromagnetic stirring was performed for 10 h until the HA was completely dispersed and evenly distributed. The configured solution was then placed in a mechanical-extrusion-deposition rapid-prototyping system. The sample was randomly printed and was then freeze-dried for 48 h. After the organic solvent evaporated completely, the samples were removed and brought to room temperature. After finishing the processing of the samples (Figures 1(b) and 1(c)), the porous scaffolds required for the experiment were obtained and were disinfected.

#### 2.3.2. Rabbit Experiments

**Figure 2:**
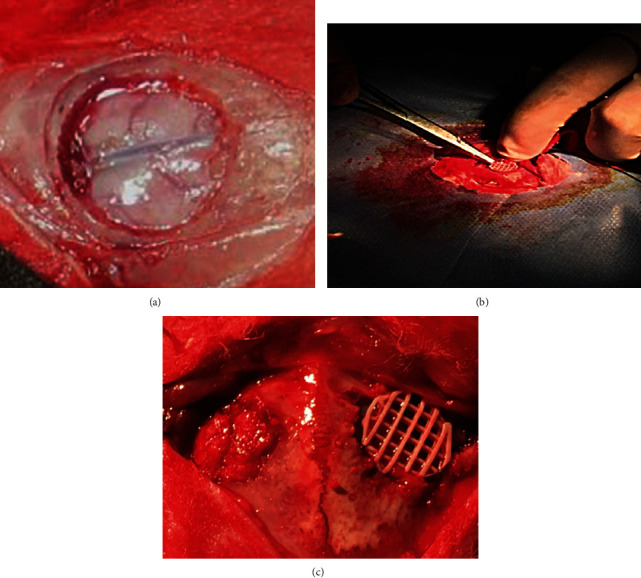
Preparation and filling of hole A and hole B.

Twelve healthy adult New Zealand rabbits were selected as experimental animals. After preoperative observations, each rabbit was weighed, skin was prepared on the head, and 0.2 ml of tachypia was injected into the ear vein. After the animal was quiet, 2% sodium pentobarbital (1 ml/kg) was slowly injected into the ear vein. The scalp, muscle, and periosteum were dissected longitudinally. After the periosteum was separated from the middle to both sides, a coronal suture and sagittal suture could be seen. Two full-thickness skull defects of about 1.0 cm in diameter each were formed by drilling holes in the parietal bone of each New Zealand rabbit (Figure 2(a)). The drilling area provided a 0.9% saline injection for cooling. Additionally, we were careful to collect the removed bone after drilling and ensured that the dura was intact and without damage. Our two drilled holes (hole A and hole B) were 0.5 cm apart from one another. In hole A, we placed 3D-printing HA-PLA composites as our experimental manipulation (Figures 2(b) and 2(c)). In hole B, we placed tamponade intraoperative autologous bone as our control manipulation. Then, the periosteum and muscles were sutured, and the skin was sutured with silk threads. An intramuscular injection of penicillin was given 400,000 units/time, twice a day, for three days to prevent infection.

#### 2.3.3. Detection and Sampling

Six experimental rabbits were sacrificed at 30 days after the operation, whereas the other six rabbits were sacrificed at 90 days after the operation. Specimens were later cut around the two holes and were fixed with 10% formaldehyde to observe the following features. First, general observations of the muscle and subcutaneous tissue attached to each skull specimen were made by removing these tissues from the surface of the skull membrane and documenting the following: the connection between the implanted HA-PLA composites and the surrounding bone; new bone formation; and degradation of the filling materials. Second, hardness testing was conducted by using a microhardness tester to measure the hardness of the specimens and that of the surrounding normal bony structures. We used a diamond indenter to specify the load pressure into the experimental samples. The pressure of the microscope cross-wire alignment pits in the eyepiece micrometer was used to measure the length of the indentation diagonal to determine the microhardness of the samples, 5 points were randomly selected in hole A, hole B, and the surrounding normal bone for measurement, namely, experimental group (hole A), control group (hole B), and normal group (normal bone). Finally, we conducted pathological observations of all the samples after they were fixed in 10% formaldehyde for 48 h and were then decalcified with neutral decalcification agent at room temperature. After decalcification, the samples were rinsed with distilled water for 8 h. Conventional dehydration, paraffin-embedded sections, and hematoxylin and eosin staining were performed.

### 2.4. Statistical Analysis

SPSS 20.0 software was used for statistical analysis. Paired *t*-tests were used for comparisons of materials at the same time points, and *p* < 0.05 was considered statistically significant.

## 3. Results

### 3.1. General Observations of Rabbits after Operations

All rabbit experiments were completed within seven days. One rabbit died unexpectedly due to anesthesia before surgery, and a replacement rabbit was included in the study sample. All other experimental rabbits were in good condition after surgery. Skin incisions healed well in the experimental animals.

### 3.2. General Observations

**Figure 3:**
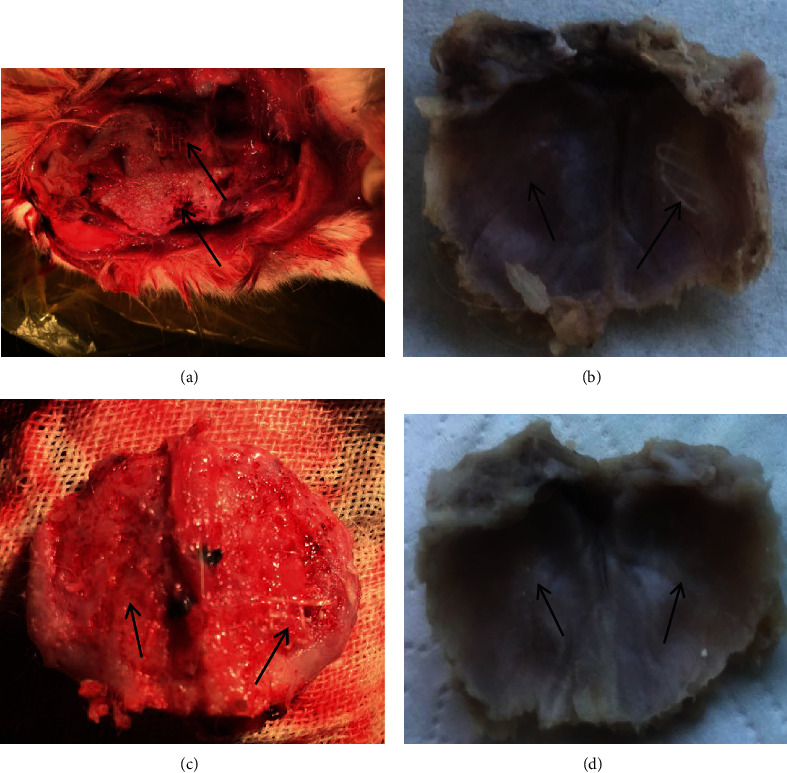
Healing of cranial defects in rabbits at 30 days and 90 days after surgery. Arrows indicate areas of cranial defects. (a) Healing of the external side of the skull defect at 30 days after surgery. (b) Healing of the internal side of the skull defect at 30 days after surgery. (c) Healing of the external side of the skull defect at 90 days after surgery. (d) Healing of the internal side of the skull defect at 90 days after surgery.

At 30 days after operations, the autologous bone powder in the cranial defects of the control group was mostly connected with the surrounding normal bone tissue, and the bone powder had mostly filled the cranial hole. After filling, the shape was uneven, but no obvious bulge or depression was observed (Figure 3(a)). Thin cortical bone was naturally connected with the surrounding normal bone on the inner side of the skull (adjacent to the meninges). In the experimental group, there was no obvious displacement of the HA-PLA complex. A small amount of new bone attachment was observed at the junction with the normal bone, and the fixation was relatively firm. A small amount of new bone and some fibrous tissues were observed in the complex grid, and no obvious uplift or depression was found in the filling of the hole. Thin cortical bone was also observed near the meningeal layer (Figure 3(b)). At 90 days after operations, the contour of the defect was dimly observed by the naked eye in the control group. The defect had been mostly repaired, and the defect was mostly consistent with the contour of the entire skull. In the experimental group, the defect was roughly observed with the naked eye to have been mostly filled with new bone tissue and fibrous tissue, forming an integral whole with the surrounding normal bone tissue (Figure 3(c)). The contour of compound could be seen on the outer side of the skull, some implants were degraded, and the new bone in the defect on the inner side of the skull was naturally connected with the surrounding tissue (Figure 3(d)). There was no obvious dural damage, epidural hematoma, effusion, or abscess in the control group or the experimental group.

### 3.3. Hardness Testing

**Figure 4:**
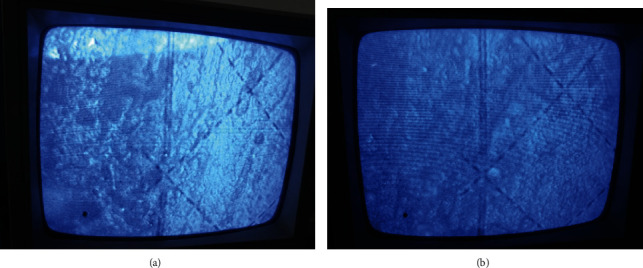
30-day hardness testing and 90-day hardness testing.

At 30 days after surgery, the microhardness of the repaired area of the 3D-printed HA-PLA complex was lower than that of the repaired area of autogenous bone (*p* < 0.01) (Figure 4(a)), while neither of these treatments reached the hardness of normal bone at this time (*p* < 0.01). At 90 days after surgery, there was no statistically significant difference in the microhardness of the repaired area of the 3D-printed HA-PLA complex compared with that of the repaired area of autogenous bone (*p* > 0.05; Figure 4(b); Tables 1 and 2).

### 3.4. Histological Observations

**Figure 5:**
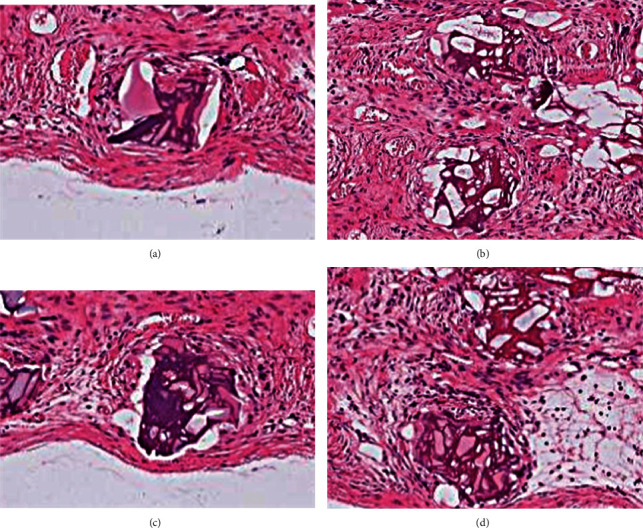
Eosin-stained sections of hematoxylin of newborn skull in the defect area at 30 days and 90 days after surgery. Images show examples at (a) 30 days after surgery for the experimental group (HEX100), (b) 30 days after surgery for the control group (HEX100), (c) 90 days after surgery for the control group (HEX100), and (d) 90 days after surgery for the experimental group (HEX100).

At 30 days after operations, the trabeculae in the experimental group were observed at 100-fold magnification under a light microscope and were found to be well formed, part of the bone trabeculae was connected, most of the bone trabeculae were arranged orderly along the direction of skull defects, and formations of new blood vessels and marrow cavities were visible (Figure 5(a)). In the control group, the trabecular thickness was good, but the orientation was irregular (Figure 5(b)), which may indicate that HA-PLA composites play a role in inducing bone repair and supporting bone cell growth. At 90 days after operations, the experimental group was observed at 100-fold magnification under a light microscope, and dense trabeculae were found to be formed and well connected. HA-PLA composites were found between the trabeculae, and bone cells and trabeculae were observed to have traveled between the materials (Figure 5(c)). This finding indicates that under the guidance and support of HA-PLA composites, bone tissue grows into the space between the HA-PLA composite. Additionally, bone units and marrow cavities were also observed. A large number of active osteoblasts were seen around the HA-PLA composite, and active osteoblasts in the HA-PLA composite were found to have an orderly arrangement (Figure 5(d)). These findings further demonstrate that the HA-PLA composite induced bone growth and provided an effective site for bone cell migration.

## 4. Discussion

Cranioplasty is a common neurosurgical procedure performed to reconstruct cranial defects. The materials used to replace bone defects have evolved throughout history. Cranioplasty materials should have the following characteristics: (1) radio-permeability, (2) infection-resistant, (3) not conductive or cold, (4) resistant to biological and physical processes, (5) extendable to accommodate and completely close the defect, and (6) inexpensive [6]. At present, commonly used cranial-repairing materials include autologous bone, titanium mesh/titanium plates, PMMA, PRRK, and hydroxyapatite [6–9]. Cranioplasty materials can be broadly divided into biological and synthetic materials. Autologous bone—due to its biocompatibility, complementarity-mutual exclusiveness, low cost, ability to grow, and ray through sexual characteristics—represents the current standard for bone repair materials. There are two common bone repair methods. In the first repair method, the original bone flap and implant can be taken as the bone graft donor area, in which the original bone flap is removed; back surgery can achieve a satisfactory appearance and represents the best choice for skull repair, but certain conditions are needed for the upkeep of the preoperative bone flap. At early stages, the original bone flap is preserved in the abdominal skin of the patient to retain survival, but this affects the quality of life of the patient and increases the risk of infection caused by the surgical site. At present, this method has been banned by some countries. Another method for bone repair is cryopreservation, but not every neurosurgery center can employ this method; moreover, clinical studies have found no statistical difference in postoperative infection rates between these two bone repair methods [9, 10]. In addition to the original bone flap, commonly used donor areas include the tibia, ribs, and hip. In defects on the lateral plate of the skull, the use of the donor area for bone repair of small skull defects has achieved promising results. However, due to the limited source of autogenous bone, the affected bone can have increased secondary trauma, prolonged operation times, as well as difficulty in shaping the defect. For patients with infection or reabsorption after cranioplasty with autogenous bone grafting, grafts with synthetic materials after removal of the graft often yield better results. Autogenous bone is widely used in the reconstruction and development of pediatric skulls. Over time, autogenous bone healing and pediatric skull development tend to be consistent and eventually form a complete whole. Such characteristics make autogenous bone the optimal repair material for this age group. However, there are still many limitations in the application of autologous bone in adults. Therefore, researchers have recently focused attention on discovering and developing more suitable repair materials. An ideal cranial repair material should imitate the characteristics of the original bone [9]. Therefore, an ideal cranial repair material would not only fill the bone defect but also act as a scaffold with certain bone inductivity and conduction to achieve the effect of bone regeneration. As an important protective barrier for intracranial brain tissue and other nerve structures, the mechanical properties of the skull also need to be considered. With the emergence of tissue engineering technology, biomimetic properties and mechanical properties of materials can be combined. Additionally, complexes formed by the combination of different substances have also been widely used.

Therefore, in order to overcome the above deficiencies, the research of medical composite materials gradually attracted attention [11]. Medical composite material is two or more kinds of biodegradable absorbent materials, according to a certain preparation method and material ratio composite, complementary advantages, and characteristics. The composite of hydroxyapatite (HA) with organic materials such as polylactic acid (PLA) may be an important way to obtain ideal bone repair materials [12]. Polylactic acid (PLA) has good biocompatibility and bone conduction properties and can be completely biodegraded. Its degradation product is lactic acid, which can enter the body's tricarboxylic acid cycle metabolic pathway, and finally transform into carbon dioxide and water [13, 14]. Hydroxyapatite has good bioactivity and biocompatibility with bone and teeth in composition and structure.

In this study, we selected the bone graft material HA-PLA, which has great similarity to human bone tissue. HA-PLA has good histocompatibility, bone conductivity, and certain porosity and strength. It has been approved by China SFDA for clinical application, and has achieved good results in the field of small bone graft in orthopedic clinic. HA-PLA material has high compressive strength and tensile strength. The interface of the two phases is well combined, and the degradation rate in vivo is relatively slow. In addition, the degradation rate of PLA was controlled by the mixing ratio of HA-PLA, the participation of PLA with high molecular weight, and the introduction of hard segment structure into PLA, so that the decline in the strength of the composite could compensate for the strength of new bone deposition. The tensile strength and compressive strength of composite materials are close to that of human skull, while the bending performance and impact toughness of composite artificial skull are obviously better than that of human skull. The mechanical strength of composite artificial skull is very close to or even exceeds that of human skull, and the mechanical strength of 4 mm thickness is equal to that of human skull. 3D-printing technology is a type of rapid prototyping technology and has been widely used in the medical field. Compared to properties of traditional manufacturing methods, 3D printing has an improved preoperative skull-defect stent material ratio, porosity, and pore-diameter ratio indicators (such as comprehensive control). Additionally, 3D printing can be combined with CT 3D-reconstruction technology to achieve accurate and personalized treatments. Therefore, in the present study, we investigated the repair of skull defects via 3D-printed HA-PLA composites.

What is the mixture ratio of HA-PLA? It is also one of the key points of this study. The pore spaces of biological scaffolds are sites for bone cell regeneration and migration and, hence, partially determine the bone regenerative capacity of scaffolds [15]. By comparing the mechanical strength of HA-PLA composites with different mass fractions, Dang et al. concluded that when the mass ratio of HA-PLA was 15 : 85, the porosity required by biological scaffolds could be satisfied and the mechanical strength requirements of tissue engineering materials could be met, while avoiding the agglomeration of HA could effectively improve the porosity of the composite [16]. Liu et al. used HA-PLA to prepare composite microspheres. With the increase of HA mass fraction, the glass transition temperature of the composites showed a trend of decreasing first and then increasing, while the decomposition temperature increased, and the thermal stability of the composites increased, which affected the waxy properties of the composites [17]. Therefore, we prepared the HA-PLA composite by using the above research characteristics, and set the macroscopic porosity of the mesh complex as 60%, in order to observe the bone regeneration of the HA-PLA implant to a greater extent on the basis of meeting a certain mechanical strength.

In the present study, it was found that at 30 days after surgery, the microhardness of the repaired area in the experimental group and the control group was lower than that in the normal bone, and the microhardness of the repair area in the experimental group was also lower than that in the control group, indicating that the osteogenesis time of bone cells induced by HA-PLA composites was longer than that of autogenous bone powder. At 90 days after surgery, there was no statistically significant difference in the microhardness of the HA-PLA complex repaired area compared with that of the autogenous bone repaired area and normal bone (*p* > 0.05), indicating that the bone regenerative ability of the HA-PLA complex was equivalent to that of autogenous bone with the extension of time. The bone trabeculae in the experimental group were well formed after 30 days of surgery in eosin-stained skull sections, and some of the bone trabeculae were connected. Most of the bone trabeculae were arranged orderly along the direction of skull defects, and the formation of new blood vessels and marrow cavity was observed. The trabecular thickness in the control group was higher than that in the experimental group, but the trabecular direction was irregular and disorderly. This finding indicates that the HA-PLA composite had a sufficient ability to induce bone cell migration. No significant difference was found between the experimental group and the control group after 90 days. Because autologous bone powder contains living bone cells, it can directly fill the defect and turn into bone, which has the function of osteoinduction and direct osteogenesis [18]. Autologous bone is still the gold standard of skull repair materials, but it is difficult to be widely used due to the limited sources and difficult preservation [19–21].

After cranioplasty, complications of different degrees may occur due to a variety of factors, such as subcutaneous blood and fluid accumulation, incision infection, and exposed repair materials. Because the skull defect in the present study was small, the repaired defect was also small, and no postoperative complications were found. More animal and clinical experiments are needed to explore whether there is a rejection reaction and discomfort of implantation following the procedures used here. In the present study, we found that the HA-PLA complex degraded itself over time, induced regeneration of bone cells, and provided a conducive site for the migration of bone cells, all of which satisfied the requirements of biological scaffolds for the regeneration of bone cells. These characteristics have certain advantages over other repair materials. The ideal repair material should have good biocompatibility, bone induction, bone regeneration, biodegradability, and mechanical properties. In the present study, HA-PLA composites exhibited the above characteristics and yielded a sufficient efficacy in terms of repairing of rabbit skull defects. However, it is necessary to evaluate a material from multiple aspects of biological activity, cytotoxicity, structural stability, anti-infectivity, and histocompatibility. The advent of 3D printing has made it possible to better repair skull defects. Through 3D printing, various materials can be made into personalized skull-defect repair prostheses that perfectly match the patient's skull, which improves the accuracy and efficiency of the prosthetic system. In addition, personalized skull-defect repair can be realized, and intraoperative risks can be reduced. However, the exploration of cranial repair materials still requires further efforts from scientists. Although HA-PLA composites have some theoretical advantages for cranioplasty, they are a long way from being used as implants in humans.

## Figures and Tables

**Table 1:** Mean values from hardness tests of the experimental group, control group, and normal group at 30 days and 90 days after surgeries.

Group	Mean (hv_0.05_)	Standard deviation	Standard error of the mean
30 days			
Experimental group	10.83480	0.976071	.180031
Control group	15.70360	1.017893	.184015
Normal group	22.09820	.605393	.128787
90 days			
Experimental group	24.44777	2.778524	.489029
Control group	23.78440	2.520406	.441904
Normal group	23.90140	1.158265	.209643

**Table 2:** Hardness values of the experimental group, control group, and normal group at 30 days and 90 days after surgeries.

Matched group		Mean difference	Standard error of mean difference	95% confidence interval of standard deviation		*t*	*p*
				Lower limit	Upper limit		
30 days							
1	Experimental group-control group	-4.8688	0.2244	-5.3278	-4.4098	-21.693	0.001
2	Experimental group-normal group	11.2634	0.1909	-11.6537	-10.8731	-59.017	0.001
3	Control group-normal group	-6.3946	0.2180	-6.8405	-5.9487	-29.331	0.001

90 days							
1	Experimental group-control group	0.6634	0.4895	-0.3378	1.6645	1.355	0.186
2	Experimental group-normal group	0.5464	0.5315	-0.5406	1.6334	1.028	0.312
3	Control group-normal group	-0.1170	0.5107	-1.1615	0.9275	-.229	0.820

## Data Availability

Data are available from the corresponding authors.
